# Cultivating Computational Thinking and Social Play among Neurodiverse Preschoolers in Inclusive Classrooms

**Published:** 2025

**Authors:** Maitraye Das, Megan Tran, Amanda Chih-han Ong, Julie A. Kientz, Heather Feldner

**Affiliations:** Khoury College of Computer Sciences, Northeastern University, Boston, Massachusetts, USA; University of Washington, Seattle, Washington, USA; Human-Centered Design and Engineering, University of Washington, Seattle, Washington, USA; Human Centered Design and Engineering, University of Washington, Seattle, Washington, USA; Department of Rehabilitation Medicine, University of Washington, Seattle, Washington, USA

**Keywords:** Computational thinking, inclusive classroom, neurodiverse, preschool

## Abstract

Computational thinking (CT) is regarded as a fundamental twenty-first century skill and has been implemented in many early childhood education curriculum. Yet, the needs of neurodivergent children have remained largely overlooked in the extensive research and technologies built to foster CT among children. To address this, we investigated how to support neurodiverse (i.e., groups involving neurodivergent and neurotypical) preschoolers aged 3–5 in learning CT concepts. Grounded in interviews with six teachers, we deployed an age-appropriate, programmable robot called KIBO in two preschool classrooms involving 12 neurodivergent and 17 neurotypical children for eight weeks. Using interaction analysis, we illustrate how neurodivergent children found enjoyment in assembling KIBO and learned to code with it while engaging in cooperative and competitive play with neurotypical peers and the adults. Through this, we discuss accessible adaptations needed to enhance CT among neurodivergent preschoolers and ways to reimagine technology-mediated social play for them.

## 1 Introduction

Computational thinking (CT)—or the ability to use concepts from computer science to solve problems—is regarded as a fundamental twenty-first century skill [6, 71]. This cognitive skillset includes algorithmic thinking, logical reasoning, breaking down complex ideas into smaller parts, and uncovering issues or “bugs” in solutions [11]. Over the last two decades, education researchers, policymakers, and industry practitioners have implemented many initiatives to promote CT among children, often in classroom settings where children work together, share ideas, and support one another’s CT development; see [3, 75] for an overview. However, a vast majority of this work has focused on non-disabled children [28, 75], overlooking the needs of neurodivergent children who process information and interact with others differently and require tailored approaches to learning [23, 63, 69, 74]. This oversight can exclude neurodivergent children from valuable CT learning experiences and peer interactions that are crucial for cognitive and social skill development. As of 2022, 95% school-age disabled students in the US attend mainstream schools alongside their non-disabled peers [22]. Thus, investigating the intersection of CT and social play among neurodiverse^1^ groups of children is critical to inform inclusive classroom practices and the design of accessible computational kits (e.g., programmable robots).

While research on improving CT among neurodivergent children remains nascent, HCI researchers have made significant strides in designing technologies to augment social play between neurodiverse children [20, 24, 47, 60, 70]. However, much of this research involves children aged six and older, leaving questions about how to support social play involving younger neurodivergent children. This gap is critical, as early exposure to socioemotional learning and CT can mitigate educational inequities [3, 62] and lay the foundation for effective education throughout a child’s academic journey. Therefore, our inquiry into CT-oriented inclusive social play is guided by two research questions:

- How do the design of computational kits and other adaptations shape neurodivergent preschoolers’ understanding of CT concepts?
- What forms of interpersonal interactions emerge between neurodivergent and neurotypical preschoolers (and adults) while playing with a computational kit?

To address these questions, we situate our investigation within two preschool classrooms involving 12 neurodivergent and 17 neurotypical children aged 3–5. Grounded in classroom visits and formative interviews with six teachers, we deployed an age-appropriate, programmable robot called KIBO [6] for eight weeks. Through multimodal interaction analysis, we found that neurodivergent children found enjoyment in assembling and manipulating KIBO’s physical components and learned basic CT concepts while engaging in competitive and cooperative play with their neurotypical peers, with mediation and scaffolding by the teachers and researchers.

Our work makes three empirical contributions to HCI [72]. First, we identified accessible pedagogical practices to deploy computational kits in preschool classrooms and derived strategic adaptations attuned to neurodivergent children’s access needs and affinities. Second, we presented a rich empirical understanding of how technologies like KIBO can foster CT among neurodivergent preschoolers, shaping their approach to problem-solving and collaboration from an early age. Third, taking the lens of the Integrated Play Groups model [73, 74], we discussed ways to reimagine the design of computational kits and practices to promote inclusive social play among neurodiverse children. Our findings align with and extend prior CHI publications that deployed publicly available CT tools (e.g., Bee-bot, MaKey MaKey, ScratchJr, etc.) among underrepresented groups, such as autistic youth (16–19 years old) [37], adults with intellectual disabilities [18], older adults [58], and Hispanic/Latino families [76]. Similar to this prior work, our contributions do not lie in the development of a novel CT tool; rather we contribute new knowledge about what forms of adaptations are needed to introduce neurodivergent preschoolers to an existing CT tool that has not been specifically designed for them, and how future CT tools and practices can be made more accessible to these children. Importantly, contrary to the prior work [1, 5, 25, 37, 79], our focus is on a younger population (aged 3–5) whose needs are shaped by both their neurodivergence and early developmental phase.

## 2 Related Work

Our research is informed by the frameworks and technologies for teaching CT to children with and without disabilities as well as social play involving neurodivergent children.

### 2.1 Computational Thinking Frameworks and Tools for Children

Brennan and Resnick [11] proposed a CT framework with three dimensions: (1) *concepts* used when creating programs (sequences, cycles, parallelisms, events, conditional, operators and data); (2) *practices* that describe how—not what—one is learning (being incremental and iterative, testing and debugging, reusing and remixing, abstracting and modularizing); and (3) *perspectives* that evolve among children while playing with computational kits (expressing, connecting, and questioning). Subsequently, Bers [6] provided a developmentally appropriate CT framework for children aged 4–9 [54], enumerating seven “powerful ideas”: algorithms (sequencing/order, logical organization); modularity (breaking up a larger task into smaller parts, instructions); control structures (recognizing patterns and repetition, cause and effect); representation (symbolic presentation); hardware/software (“smart” objects are not magical but human-engineered); design process (editing/revision); and debugging (identifying and solving problems).

Over the years, numerous tools have been developed to teach children these CT concepts; see [3, 75] for an overview. These include digital kits (e.g., ScratchJr [21] where children can drag- and-drop graphical blocks to create interactive stories), physical kits (e.g., KIBO [6] which comprises tangible robots controlled with programming blocks), and hybrid kits (e.g., Strawbies [31], a tablet game where wooden tiles are used to control virtual characters). To guide the design of child-centric computational kits, Resnick and Silverman [56] posited metaphorical principles, namely *low floors* (short learning curve for novices); *high ceilings* (accommodating increasingly complex projects); and *wide walls* (many paths for self-expression). Alper et al. [2] re-elaborated these principles to support the needs of disabled children: *low floors with ramps* (e.g., nonvisual cues for blind children); *high ceilings with tall ladders* (e.g., scaffolds that let children with intellectual disabilities progress at their own pace); *wide walls with frames of interest* (e.g., allowing depth in specific topics that autistic children may prefer over exploring the breadth of possibilities); and *reinforced corners* for children who may need additional support to thrive at the widest walls, highest ceilings, and lowest floors.

### 2.2 Programming and Computational Technologies for Children with Disabilities

Recent surveys highlight various accessibility issues in commercially-available and research-led computational kits [28, 75]. In parallel, HCI and accessibility scholars have started investigating ways to aid disabled children in learning programming and computational concepts. For example, Milne and Ladner [42] incorporated audio cues and screen reader support on a touchscreen app to adapt block-based programming for blind children. Another popular strategy involves building tangible interfaces (e.g., StoryBlocks [36], Torino [44], ACCembly[57], TIP-Toy [4]) that enable blind children to learn computational concepts by manipulating physical objects, often in collaboration with sighted peers. Researchers also conducted co-design workshops where blind and sighted children collaboratively built voice user interface apps [40], multisensory storytelling tools [15, 39], and tangible games using robots [38, 45, 46].

Compared to the above studies involving blind children, limited work has examined ways to enhance CT among neurodivergent children. Zubair et al. [79] identified challenges adolescents with autism and learning disabilities (13–14 years) encountered on Scratch [55]. Others introduced computational robots like KIBO, Bee-Bot, or Magic Cubes to children with autism (aged 6–14 [1] or 16–19 [37]) and Down Syndrome (aged 7–19 [25] or 5–12 [5]), where children showed sustained interest in playing with the robots, although their understanding of CT concepts were limited. While these studies take an important step towards inclusive education, many open questions remain around how to best support preliterate neurodivergent children (aged 3–5) in learning CT concepts.

### 2.3 Social Play Technologies for Neurodivergent Children

While promoting CT among neurodivergent children remains underexplored, a growing body of research investigates technologies for social play among neurodivergent children [34, 59]. Researchers found that tangible interfaces like programmable construction kits [19, 20] or sonic e-textile interfaces [47, 48] can increase cooperative play among autistic children. Others noted that tablet apps [9, 30, 64] and tabletop games [52, 78] can augment social-emotional skills through collaborative engagement between neurodivergent and neurotypical playmates.

Much of the earlier research in this domain aims to teach neurodivergent children to replicate neurotypical behaviors [65], given their play patterns are considered undesirable or purposeless. Recently, however, researchers have started shifting from this deficit-oriented narrative. Wolfberg [73] put forth the Integrated Play Groups (IPG) model emphasizing the need for enhancing mutual understanding among neurodiverse children and flexible scaffolds for all to play. Wilson et al. [69, 70] developed a “Co-Design Beyond Words” approach in which minimally-verbal autistic children aged 5–8 exhibited joint attention, turn taking, and imitation through the design of playful tangibles. Frauenberger et al. [23, 24] posited that co-designing with neurodiverse children requires careful mediation of individual and group spaces, balancing between openness and structure, allowing solitary time for emotional regulation, and nurturing constructive disagreements. Drawing on the notion of “expanded proxy” [41], others explored how neurodiverse children (aged 5–12) co-designed social play technologies for a ‘stuffed animal friend’ with personality traits of a neurotypical or neurodivergent child [14, 51]. We adopt this broader view of neurodivergence but focus on a younger population (neurodiverse preschoolers aged 3–5) and examine how social play unfolds through children’s interaction with a computational robot.

## 3 Context of Study

### 3.1 Inclusive Preschool Classrooms

Our research took place in two preschool classrooms within an early childhood education and research center in a major US city. One classroom (A) had 14 children and the other (B) had 15; all aged 3–5. Six children in each class were neurodivergent. The rest were neurotypical. Table 16 in the Appendix reports participating children’s details (all names are pseudonyms). Each classroom had one lead teacher, 3–4 teaching assistants, and several volunteers who assisted with classroom activities. Speech-language pathologists and occupational therapists performed consultative services weekly with selected children according to their Individualized Education Program (IEP) [8]. Prioritizing both literacy and socioemotional skills development, the classroom curricula included group learning and “free choice” times for art, craft, and play.

With approval from the Institutional Review Board of the University of Washington and the school staff, our study began with the first author (Das) conducting four hours of observation in two classrooms in March 2023. These early visits provided insights into the classroom environment and children’s interests, informing our choice of technology for the deployment study.

### 3.2 Technology for Deployment: KIBO

**Figure 1:**
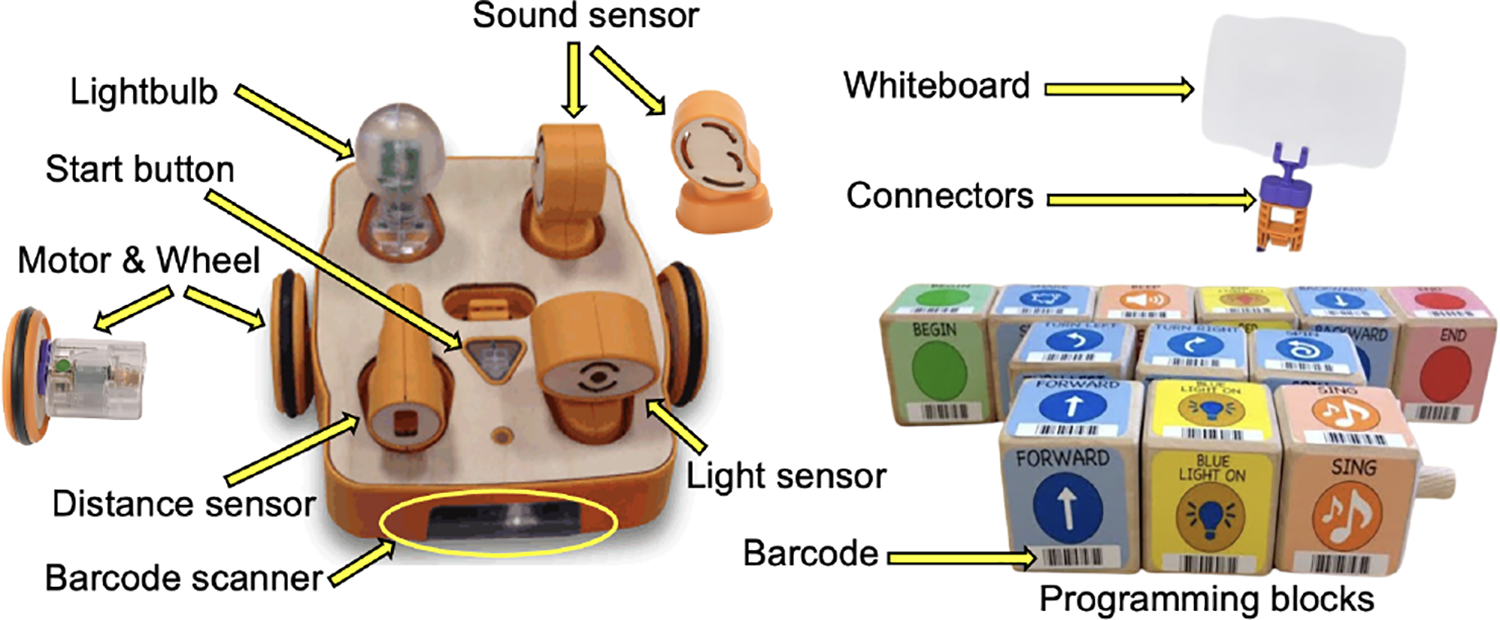
KIBO kit [35]. Motor, wheel, programming blocks, sensors, lightbulb, and whiteboard are annotated.

From classroom visits, we learned that the neurodivergent children enjoyed playing with physical materials and sensory toys rather than digital apps. Hence, we chose KIBO [35], a screen-free programmable robot that has been shown to encourage children as young as three years old in creative coding [3, 6]. Each KIBO kit contains easy-to-connect construction materials including a robot body, wheels, motors, lightbulbs for output, sensors for detecting sound, light, and distance, and a whiteboard and flagpole for decoration (Figure 1). The robot can be programmed to move using wooden blocks interlocked sequentially and scanned with an embedded barcode scanner. Each program must start and end with a Begin and an End block. Other blocks represent various actions of the robot, e.g., moving forward/backward, turning left/right, shake, spin, beep, white/blue/red light on, etc.

## 4 Formative Study

To understand inclusive classroom practices, we conducted one-on-one remote interviews with three teachers from each classroom (5 female, 1 male). Lead teachers in Class A and Class B had been working at the school for 7 and 2 years respectively, while teaching assistants had 6 months–5 years of experience at the school.

### 4.1 Method

Our semi-structured interviews began with inquiring how teachers supported IEP goals and access needs of neurodivergent children, how they mediated social play and learning activities, and if/how STEM activities were incorporated in the curriculum. Next, we showed the teachers a brief video of KIBO with a high-level overview of our session plan to seek their feedback on adapting it to existing classroom practices. Each interview lasted about 60 minutes, was video-recorded, and later transcribed. We compensated teachers with a US $20 gift card.

We analyzed interview data following the reflexive thematic analysis method [10]. Taking an inductive coding approach, two researchers independently read and open-coded three transcripts each, and Das reviewed all coded transcripts. We met weekly to discuss the codes and developed themes through multiple iterations.

### 4.2 Findings

Below we detail various adaptations and pedagogical strategies teachers implemented to support neurodivergent children in learning and play alongside their neurotypical peers.

#### 4.2.1 Visual Aids.

Visual aids were essential for neurodivergent children to express themselves and communicate with others. Some children used tablet-based augmentative and alternative communication (AAC) apps to form a message by pressing buttons or pointed at pictures of words that are part of their frequently used vocabulary on printed “core word boards” [12]. Teachers also prepared step-by-step graphic instructions for specific activities. T1 explained, *“We always make a visual sequence… pictures of what we’re actually doing and they can follow along… Ways that we can take away the [requirement of] speech and have them point… using their talkers (AAC apps).”*

#### 4.2.2 Adult Modeling.

When learning new concepts, neurodivergent children often faced difficulty in grasping verbal instructions. Hence, teachers combined speech with nonverbal cues, such as gestures, postures, facial expressions, and body movement so that children could replicate their actions and directions. Eventually, children learned to perform the actions by themselves; however, the *“modeling”* approach early on helped them process new information without feeling overwhelmed. T1 elaborated, *“If you’re like ‘Put this in there,’ they sometimes don’t know what you’re talking about. Showing them exactly how to do it… [with] gestural prompts… and then giving them a turn [helps].”*

#### 4.2.3 One-on-One Support.

Teachers emphasized the significance of accommodating children who need *“intensive support”* (T2) to adjust their learning pace with task complexity. For these children, *“one-on-one with a teacher can be really helpful”* (T1) to learn the necessary concepts or skills prior to engaging in a group activity. Such *“pre-teaching”* ensures that neurodivergent children are *“familiar with the vocabulary, materials, and the different ways to use it”* (T2) ahead of time so that they do not fall behind when the actual activity occurs with their neurotypical peers. One-on-one support also helps these children manage desensitization breaks and *“finish the task… after coming back”* (T1).

#### 4.2.4 Nudging and Scripting for Cooperative Play.

While designing play routines among neurodiverse children, teachers consider children’s developmental phase and IEP goals and scaffold accordingly. This may involve nudging children who are used to solitary play to explore *“simple one-on-one interactions”* (T4) with peers, while encouraging children who are comfortable in cooperative play to help their peers accomplish a task together [63]. T4 explained, *“Sometimes we ask kids [who can read], ‘Oh, can you read this book for your friend? He doesn’t know how to read, but he can express it with pictures.’ So they get engaged together.”*

Occassionally, a neurodivergent child may have *“a hard time verbalizing what she really wants from other kids”* (T1). Neurotypical children also may not understand how their neurodivergent peers express their thoughts and how to communicate with them in an accessible manner. To help children *“jump-start conversation”* with peers, teachers *“give them scripts to follow”* [63]. T2 elaborated, *“Sometimes they go and grab something from the kid they want to play with. So you can give them phrases [to say] like, ‘Can I play with you?’ or ‘What are you playing with?’… And what they can do after they get an answer from their peer—‘Yes, let’s play’ or ‘Not right now’.”* Additionally, as a form of social reward, teachers articulate *“positive praise”* when children engage in collaboration. T1 recalled praising a neurodivergent child to *“validate that she was able to do something that was trickier for her… like, ‘Wow, I noticed you asked your friend for a turn… That was a great job!’”*

#### 4.2.5 Flexible Scheduling.

Since many neurodivergent children have limited attention spans, teachers emphasized the need for flexible scheduling and enabling different modes of engagement (e.g., direct play versus onlooker behavior [60]) while introducing a new toy. T1 explained, *“We have a few students who are a little apprehensive of trying new things. So at the beginning, it might just be them observing for a few minutes. Other students, however, would jump right in and learn it pretty quickly. So it could be 5 minutes for some kids, and other kids could go for 20 or more.”*

#### 4.2.6 Balancing Agency with Concise Options.

For neurodivergent children, being able to decide how and when to participate in certain activities is crucial to learn emotional self-regulation and to have *“a sense of control over the situation.”* T3 elaborated, *“It’s not like a teacher is forcing [children] to do whatever. They have the power to make that choice and it makes them feel better. There’s a much higher rate of success when they have a say in what the next steps are going to be.”* However, sometimes neurodivergent children feel overwhelmed if they need to make decisions from a wide variety of possibilities. When handling such confusing situations, teachers provide several *“safe”* options for children to choose from. T3 said, *“If there’s a student who has no idea what’s going on, shrinking it down to just ‘this or that’ options is the most basic level you can do.”*

#### 4.2.7 Facilitating Personalized Play.

While designing classroom activities, teachers take into account children’s interests and affinities [63] including their favorite characters, objects, hobbies, and more to make their learning process enjoyable and engaging. For instance, teachers incorporated shapes and numbers into activities for Wyatt who *“is very fixated on numbers and really likes to line up shapes in a specific way”* (T6).

Overall, teachers adapted various strategies attuned to children’s needs and aptitudes to foster an inclusive learning environment for children across all abilities and developmental stages.

## 5 Deployment Study: Method

### 5.1 Developing Session Plans

Drawing on the CAL-KIBO-PreK curriculum [7] designed for pre-kindergarten children and the insights from our formative study, we prepared a session plan for our target population of neurodiverse preschoolers. We selected a subset of the learning objectives from [7] that were feasible to cover without disrupting the classrooms’ curricular priorities (see Table 15 in the Appendix) and made the following adaptations to support neurodivergent children.

- We created visual aids (Section 4.2.1), including printed sheets showing pictograms of KIBO components with large text and enumerated instructions for the core activities, such as how to assemble KIBO and how to make a new code. Although the KIBO kit included an instruction sheet, it was laden with extensive information in small fonts and low color contrast, making it inaccessible to neurodivergent and preliterate preschoolers.
- To provide children with concise options (Section 4.2.6) for questions we would ask, we prepared pictograms of probable answers (Section 4.2.1), for example, a sheet with images and text denoting ‘yes’, ‘no’, and ‘maybe’ and another sheet with ‘fun’, ‘boring’, and ‘not sure’ to indicate how they felt about an activity.
- We anticipated that learning to scan the barcode within a short timeline might be a *“big hurdle”* (T5) for neurodivergent preschoolers. Hence, we decided to scan the code ourselves but asked children to *“help”* us scan by holding the robot so that they could model after us and feel being a part of the activity (Section 4.2.2).
- We invited a teacher to join the sessions that involved neurodivergent children who needed *“pre-teaching”* (Section 4.2.3). Because of their deeper knowledge of the children’s needs, teachers were better positioned to help children understand and manipulate KIBO and communicate their emerging needs to us.
- To encourage social play, we fluidly nudged children to share materials, take turns, and help each other and complimented them for working together (Section 4.2.4).
- Although we initially planned each lesson to last about 25–30 minutes, we adjusted the duration depending on the children’s mood and attention in the moment (Section 4.2.5).
- We learned children’s favorite animals, characters, or objects from the teachers ahead of time and printed those out for children to use for decorating KIBO (Section 4.2.7).
- We carefully managed our space and setup to minimize distractions and stimulants for neurodivergent children. Typically, all attendees (i.e., children, researchers, a teacher) sat in a circle with the KIBO positioned at the center. However, we hid programming blocks and components that were not immediately needed out of children’s sight and brought in the center only those materials that would be needed at a given time.

### 5.2 Data Collection

We brought a KIBO kit twice per week on average to the classrooms (four consecutive weeks in each class) in May–June 2023. Sessions occurred with 2–3 groups of children during “free choice” times each day with parents’ permission. We coordinated with the teachers to invite a pair of children with their assent to attend each session. We held 23 sessions in Class A and 15 in Class B (total 38). Every child attended at least one session, with the highest count being 7 in Class A (median 3) and 6 in Class B (median 2). Some children continued to play after our planned activity ended while others did not stay for the full activity, resulting in session duration ranging between 12–39 minutes (mean 22.6).

During the “free choice” time, several play-stations were set up in different corners of the classroom. Our station was set up in such a corner which was secluded but not entirely out of view from the rest of the class. Therefore, children who were not invited in a particular session sometimes sat beside the invited children to watch them play or joined them near the end of the session. The first author, Das facilitated the planned activities, while a research assistant managed video recording and took field notes. Thus, Das actively participated in the sessions, not just as an observer but also as an interactant in the socio-material context of the study.

We recorded the sessions, generating about 14.32 hours of video data. Two cameras captured the play area from two different top views to record children’s interactions with KIBO and with each other. We sent a US$20 gift card to each child’s parent and a set of educational toys (of up to US$100 total) to each classroom.

### 5.3 Data Analysis

We analyzed video data following multimodal interaction analysis, a method from the learning sciences used to assess learning in complex environments [17, 49]. This method allowed us to uncover how neurodivergent children (especially those who were minimally- or nonspeaking^2^) used rich, nonverbal cues to communicate with others and how adults incorporated embodied adaptations to support their needs—insights that may not have been fully captured without a detailed video analysis. We drew from prior research that used interaction analysis to study embodied communicative practices of neurodivergent [62], aphasic [27], and blind people [67] with their non-disabled conversation partners.

Four researchers transcribed non-overlapping subsets of the entire video corpus, while Das reviewed all transcripts and video side-by-side. We open-coded the transcripts, supplementing with field notes and photos captured during deployment, and revised the codes through weekly group meetings. Given our research focus, we thoroughly analyzed 32 play sessions that involved at least one neurodivergent child. The remaining six sessions (including only neurotypical pairs) underwent cursory review but were excluded from deeper analyses. While some neurodivergent children attended only 1–2 sessions, they demonstrated interesting interaction with KIBO and others; and thus were included in our analysis. We considered interaction as multimodal, embodied, and situated [26, 27] and looked for salient interactions that captured, for example, a neurodivergent child pointing to a visual aid, two children putting blocks together, teachers providing hand-over-hand guidance, etc. Although we took short notes on all episodes of such interactions, we produced detailed memos for unique “hot spots” strategically selected for deeper analyses [17]. Through iterative comparison of codes and data, we constructed three overarching themes that described how neurodiverse children engaged in coding and social play with each other, with adults, and around KIBO.

## 6 Deployment Study: Findings

Our findings demonstrate that neurodiverse children found enjoyment in manipulating KIBO’s physical components and learned to code, while navigating conflicts and negotiating cooperation with their playmates. Below we present our findings using a narrative-oriented approach with illustrative transcripts [17] depicting interactions between neurodivergent and neurotypical children, teachers, and/or the lead researcher, Das around KIBO.

### 6.1 Assembling and Decorating KIBO

Four neurodivergent children (Bryce, Sierra, Violet, and Cooper) demonstrated sustained engagement with KIBO and completed most of our lessons, starting from introduction to KIBO to making new codes that required higher-level conceptualization of CT concepts. Additionally, four minimally- or nonspeaking children (Hazel, Kevin, Aaron, and Wyatt) attended three sessions each. Regardless of their level of engagement, most neurodivergent children exhibited interest, and at times heightened enthusiasm, about assembling KIBO components and decorating it [1].

The physical, tactile, and kinesthetic affordances of KIBO helped children understand and tinker with this new technology. Yet, for minimally- or nonspeaking children, our pre-planned and in-the-moment adaptations were instrumental in making the interaction with KIBO accessible and enjoyable. Below we present two examples that can guide educators in incorporating similar construction materials (e.g., vehicle assembly kit, LEGO) in neurodiverse classrooms. Table 1 demonstrates a vignette involving Avi, an autistic and nonspeaking child who explored KIBO through repetitive and rhythmic movements, e.g., shaking and banging components to assess their weight, rigidity, and sound (Lines 1, 5, 6, 10, 12)—actions that are likely part of his ‘stimming’ i.e., self-stimulation activities for sensory regulation [61, 66].

In Line 3, Avi attends to Das’s verbal instruction and non-verbal gesture to insert the motor into the motor slot. However, he does not understand Das’s deictic reference (“it”) to the motor (Line 2). He instead tries to place the wheel on the motor slot and then starts banging the motor and the wheel, possibly to see if they can be connected or what sounds they make (Lines 5–6). Later, T2 offers to help and Avi hands her the motor and the wheel (Lines 7–8). T2 inserts the motor into its slot without fully securing it. She encourages Avi to model after her by intonating *“I’m gonna push it”* (Line 9). Avi, however, pulls out the motor from the slot (Line 12), possibly because as a *“gestalt language processor,”* he may have confused the words ‘push’ and ‘pull’ [29]. As T2 clarifies the misunderstanding, Avi follows through her instruction and pushes the motor into the slot (Lines 13–14). T2 repeats the process by placing the wheel on the motor’s axle and asking Avi to “push” (Line 16). This time, Avi readily pushes the wheel while T2 holds it in place (Line 17).

This vignette illustrates Avi’s continued interest in KIBO as evident by his fiddling with the motor, wheel, and robot body. However, in-the-moment scaffolding from T2 played a crucial role for Avi to successfully assemble the robot. T2 assessed exactly what part of the task was difficult for Avi (e.g., deciding which component fits what slot) versus what he could easily accomplish (e.g., pushing the motor when inserted in place) and provided support accordingly.

Like assembling KIBO, decorating it became another enjoyable avenue of pretend play among children [59]. The vignette in Table 2 centers around Sierra (autistic, minimally-speaking) who often pretends to be a superhero wearing a red cape. When it is time to decorate KIBO, teacher T1 draws Sierra’s attention to the picture of a female superhero in our materials (Line 1). Sierra excitedly says *“Super Sierra!”*, selects this picture to decorate KIBO (Lines 2–4), and claps in joy after finishing her decoration (Lines 5–6). Next, Das probes her to imagine a new name for the robot, where it lives, and what it likes to do. At this point, Sierra gets distracted and does not immediately respond (Lines 9, 12). T1 provides linguistic cues to grab Sierra’s attention and converts Das’s open-ended questions to fill-in-the-blanks (Lines 7, 10) and multiple-choice exercise, making it easy for Sierra to formulate responses (Lines 13–16).

This example shows that incorporating materials catered to neurodivergent children’s interests can enhance their playful engagement with robots, like other toys [63]. Simultaneously, linguistic scaffolds coupled with visual aids can draw and sustain neurodivergent children’s attention on the activity, especially for those who tend to zone off frequently. Further, providing concise options can make the wide open imaginative space more manageable for neurodivergent children and help them easily formulate and communicate their thoughts.

### 6.2 Learning to Code with KIBO

Over the course of our deployment, several neurotypical and neurodivergent children demonstrated understanding of fundamental computational concepts, as we detail below.

#### 6.2.1 Understanding the Meaning of Coding Components.

The visual aids we created including pictograms and step-by-step instructions helped children grasp the meaning of KIBO’s coding components, e.g., which blocks mapped to what actions and the functions of the sound sensor and the lightbulb output. KIBO’s default symbolic representations also provided helpful affordances for children to remember the coding components by their colors (e.g., Begin block is green, End block is red), shapes (e.g., sound and light sensors resemble ears and eyes), and symbols (e.g., Spin block has a circular arrow). The vignettes in Table 3 involving Violet (language delay) and Mila (neurotypical) demonstrate this.

First, Das probes if the children remember which blocks they had used before to begin and end their code. Mila and Violet respectively pick up the correct blocks i.e., Begin and End (Lines 1–2). Das encourages them to choose more blocks to create a new code. Violet responds, *“I want Beep”* and points to the corresponding block, demonstrating that she understands its function (Line 4). Following this, Das introduces the sound sensor using the metaphor of an ear (Line 5). When prompted, Mila responds that the sensor looks like an ear (Line 6). Meanwhile, Violet gets distracted. Das attempts to draw her attention and gives her options to agree or disagree with whether the sensor resembles an ear. Violet concurs by nodding and pointing to the ‘yes’ picture on the sheet (Line 7). Das explains that sensors help KIBO to listen to sounds, to which Violet nods (Line 8), signaling that she has understood the explanation.

At the end of the session when Violet and Mila have already created a code using the Wait-for-Clap block and the sound sensor. Das queries whether they remember what they have used to make KIBO listen to the sound, to which Violet responds “ear” holding the sound sensor (Line 10). She also correctly recognizes the Wait-for-Clap block as the one needed to make KIBO wait for the sound input (Line 11). Taken together, these snippets illustrate how the visual aids helped neurodivergent children like Violet in learning symbolic representations and communicating with others.

#### 6.2.2 Creating New Code Sequences.

Throughout our sessions, we observed that attaching one block to another came naturally to most children due to the blocks’ implicit affordances (i.e., the peg of one block goes into the slot of another). Many children enjoyed the manual process of connecting blocks (e.g., constructing a vertical tower), sometimes without comprehending the underlying coding concepts [1]. However, over time, several children learned to create new code sequences to control KIBO’s actions, as Table 4 shows with vignettes involving Sierra (autistic, minimally-speaking).

In the first snippet, Sierra has already put together a code sequence with a neurotypical peer following Das’s instructions. Later, she notices another block lying behind Das and wants to attach it to her code (Lines 1–2). Before attaching this new block, Sierra takes out the End block from the current sequence (Line 3), inserts the new block (Line 5), and then reconnects the End block (Line 6). She does this part on her own without any adult guidance, indicating that she has learned from the previous interaction the order in which a code sequence should be started and ended.

In the second snippet, Sierra immediately starts tinkering with the four blocks Das has placed before her (Lines 7–8). She independently connects the blocks to create a new code and reads it out loud when prompted (Line 10). Collectively, these snippets illustrate how Sierra learned to create new codes for programming KIBO over the course of our sessions.

#### 6.2.3 Deducing Cause and Effect.

Both neurodivergent and neurotypical children were able to understand the concept of cause and effect through their interaction with KIBO. Below we present two vignettes where children demonstrate their understanding of the if-then conditional structure by reasoning when a code sequence including the Wait-for-Clap block executes (and when it does not).

Table 5 shows Bryce (GDD) and Shiloh (neurotypical) trying to execute a code they have created together. The code starts with the Wait-for-Clap block which works in conjunction with the sound sensor: *If* there is a sound near the sensor, *then* KIBO will execute the actions after the Wait-for-Clap block. Unless the sensor detects a sound, KIBO will not move. Here, Shiloh presses the start button twice but nothing happens (Line 1). Das prompts the children to think why KIBO is not moving (Line 2). Bryce responds, “Clap!” and starts clapping repeatedly (Line 3), indicating that he has deduced the *cause* behind KIBO’s inaction i.e., *effect*. However, Bryce’s claps are soft and farther away from the sensor. Das clarifies that they need to clap closer to the sensor to trigger it (Line 4). Shiloh immediately follows this instruction and reconfirms his understanding of KIBO’s inaction, saying *“He’s waiting for your clap”* (Line 5).

In contrast to Bryce and Shiloh, who were strongly enthusiastic about KIBO, the snippet in Table 6 includes Aaron (autistic, nonspeaking) who mostly participated as an onlooker and had not shown much interest in manipulating KIBO in previous sessions. Here, Aaron joins us after another group has left creating a program with the Wait-for-Clap block. This program is still uploaded in KIBO when Das demonstrates KIBO’s action to Aaron who attentively observes it moving (Line 1). Unlike the previous sessions, the movement of KIBO here has grabbed his attention, as the teacher, T7 confirms (Line 2). Das presses the start button and claps near the sound sensor to make KIBO move. Aaron readily emulates Das and claps repeatedly (Line 3), for which T7 praises him as a form of positive reinforcement. She then guides Aaron to clap near KIBO (Line 4). When it stops after running the code, Aaron presses the start button to make KIBO move again (Line 6). This snippet eluci-dates that despite limited engagement with KIBO, Aaron was able to deduce (by observing Das) that pressing the start button and clapping will activate KIBO.

#### 6.2.4 Identifying Errors in a Code.

Even within our short timeline, children who attended repeated sessions were able to grasp the core notion of debugging. While they were not always successful in identifying problems, they understood that errors might occur in the coding or scanning process for which KIBO would not execute all the programmed actions. In one session, after connecting a series of coding blocks, Bryce (GDD) handed Das the robot and asked her to *“scan it.”* After scanning was complete, he pressed the start button, saying *“Let’s try it out. Let’s see what it does.”* This alludes to Bryce’s understanding that a new code needs to be checked to assess whether it runs as expected or not.

As another example of how children identified problems in code, we turn to the vignette in Table 7 involving Cooper (autistic) and Hugo (neurotypical). When Das prompts the children to assess if KIBO has executed every action in their code, Cooper checks the sequence of blocks and responds that it has not performed the action for the Begin block (Lines 2–3). This is not surprising; in other sessions, Bryce and Shiloh also found it difficult to conceptualize the coding blocks that did not demonstrate one-to-one observable KIBO actions (e.g., Begin and End). To clarify this confusion, Das explains that the Begin block starts the code (Line 5). To further help the children visually correlate the blocks with KIBO’s actions, Das points to individual blocks in the sequence as KIBO performs the corresponding actions (Line 7). At this point, Cooper first (and then Hugo) say that KIBO has not done the Beep action (Lines 8–9). In reality, KIBO has Beeped but skipped Spinning, which was after the Beep block. This mistake was understandable because the Beep was difficult to hear amid the noise in the classroom. Thus, from the children’s perspective, they may have indeed been able to detect the block for which they did not notice KIBO perform an action.

Recognizing the children’s difficulty in hearing the Beep, Das probes them to try again (Line 10). This time, in addition to visually pointing to the blocks, Das vocalizes the Beep sound as KIBO performs the action (Line 11). When prompted to answer which block’s action KIBO has skipped now, the children do not respond immediately. Das rephrases the question in a yes/no format and queries if KIBO has executed the Spin action (Line 12), to which Cooper readily says, “No” (Line 13). Thus, providing concise options may have helped Cooper to feel confident to answer when detecting a problem was difficult amid surrounding noise.

These sequences indicate that neurodivergent children understood that errors might occur in their code. Although they could not always correctly detect the ‘bugs,’ with step-by-step visual reference and scaffolding, they were able to identify mismatches between their code sequence and KIBO’s directly observable actions.

#### 6.2.5 Analyzing Robot Hardware.

During the introductory lessons, when we asked children questions about which picture is a robot or not, they used to respond by guessing. Over time, they started to reason through why KIBO could be considered a robot, when and how it worked, and what it resembled, as shown in Table 8. Children also engaged in thinking about how a sequence of instructions (i.e., code) can be transferred to a hardware (i.e., the robot). Moreover, although we did not teach children barcode scanning to keep our lessons simple, some neurodivergent (Sierra, Bryce) and neurotypical children (Roy, Liam, Wanda) picked up on our scanning moves and attempted to scan code sequences on their own, indicating their curiosity about KIBO’s hardware.

Table 8 shows that both Bryce (GDD) and Shiloh (neurotypical) can easily recognize the motor, wheel, and KIBO body (Lines 3–12) based on their prior interactions with it. Further, they engage in symbolic play [60] while discussing KIBO hardware. Shiloh describes the wheel as KIBO’s arms and the lightbulb as a leg (Lines 2, 12). Bryce, however, argues that the wheels are legs (Line 13). Shiloh tries to correct him, clarifying that those are “wheels” (Line 14). Next, Bryce describes KIBO as a car and Shiloh adds that it is a *“car robot”* (Lines 15–16). When Das queries whether the children feel KIBO can think on its own, Shiloh responds negatively (Line 18). Bryce explains the reason, saying *“It doesn’t work yet”* (Line 20), potentially because earlier he has noticed that KIBO has not moved after he pressed the start button (Line 10). This indicates Bryce’s understanding that a robot cannot function on its own without the code i.e., instructions that programmers provide. Shiloh further elaborates that they can make KIBO move and dance by scanning coding blocks (Line 22), as they had made the robot dance Hokey Pokey in a previous session.

### 6.3 Competition and Cooperation around KIBO

Our analysis revealed rich competitive and cooperative exchanges between neurodiverse pairs, ranging from contesting each other for the control of KIBO, working through (sometimes physical) conflicts and negotiating turn taking to helping and teaching each other coding steps and expressing conviviality and a sense of joint ownership. Below we present illustrative vignettes to exemplify how our lessons with KIBO consistently structured children’s social play, with or without adults’ direct mediation.

#### 6.3.1 Growing and Correcting Misconceptions about Neurodivergent Peers.

We observed a few instances when neurotypical children developed misconceptions about neurodivergent playmates or did not want to adapt to their needs if they required more time in completing tasks. On one occasion, Sierra (autistic) and Liam (neurotypical) paired up to create a new code. Liam loved playing with robots and had previously called himself *“a total engineer”* after assembling KIBO. Sierra also expressed enthusiasm saying *“I want to play with KIBO,”* but she got repeatedly distracted by other materials near the play area. Das tried to refocus Sierra’s attention by holding images of the coding blocks in front of her and probed her to select one that would make KIBO shake. Liam, however, grew frustrated waiting for Sierra’s turn to finish, since he wanted to add more blocks. He commented, *“She’s not really good at this.”* This shows that competing for technologies like KIBO may produce and reify neurotypical children’s misunderstanding about their neurodivergent peers’ computational skills, especially if their learning paces, styles, and attention spans are misaligned.

Similarly, Table 9 shows how Owen (neurotypical) gets impatient while playing with Avi (autistic, nonspeaking) who requires more time to assemble KIBO components. At first, Avi explores a motor and a wheel by shaking them, which is his preferred method of interacting with new objects (Line 1). Owen, however, is eager to attach the motor to a wheel he is holding (Line 2). Teacher, T2 intervenes and explains to Owen that Avi is processing the motor’s properties in his own way (Line 3). As Avi tries to connect the motor to the wheel but struggles to do it and resumes shaking them, Owen grows more impatient, commenting that Avi *“does not know how”* to do the task (Lines 4–5). When he attempts to take over, T2 steps in and nudges him to *“do it together”* (Lines 6–8). Owen, encouraged by T2’s nudging, exhibits more willingness to work with Avi (Line 9). Later, he even rejoices when Avi successfully places a lightbulb on KIBO modeling after him, saying *“I helped him!”*

This interaction exemplifies the importance of adult intervention to ensure that both neurodivergent and neurotypical children get equitable opportunities to play. Especially for computational kits, such intervention becomes crucial to prevent robot-enthusiast children from dominating the play over others who need more time and guidance to master these complex toys.

#### 6.3.2 Navigating Competition and Conflicts around KIBO.

Unsurprisingly, introducing KIBO created occasional conflicts between children around getting a hold of the new robot. Conflicts between children are common during social play. However, no children in our study had previously interacted with programmable robots in their homes or at school, likely due to the high expense or niche nature of these kits, making children fight over KIBO more often than other toys.

The following vignettes involve Hazel (Down Syndrome, minimally speaking) and Roy (neurotypical), both of whom expressed strong desire for playing alone with KIBO. Sporadic instances of competition and cooperation were woven throughout the interactions between this pair. In one instance, they shared the flagpole and the connector with each other and worked together to attach those pieces, as Das mediated turn-taking between them. Hazel sought praise and approval of this collaborative effort from Das, saying *“That good?”* However, this brief collaborative period was followed by a fight around KIBO, as detailed below.

In Table 10, Hazel follows Das’s instruction to model after Roy and places a lightbulb on one of KIBO’s four ports (Lines 3–4). However, Roy takes out the lightbulb Hazel has placed and inserts it in a different port (Line 5). The children get into a fight to grab the lightbulb, with Hazel re-inserting it on the port where she had placed it before (Lines 6–9). Here, both children are eager to show their mastery with KIBO. On one hand, Roy (incorrectly) assumes Hazel’s placement of the lightbulb to be wrong. Likewise, Hazel is irked by Roy’s (unsolicited) inference to her work. Similar conflict arises when they compete to get a hold of the sensors (Lines 10–12). Although each of them successfully places one sensor on KIBO (Lines 13–14), Hazel takes out the light sensor Roy has inserted and keeps it away from his reach when Roy tries to retrieve it (Line 16). Hazel may have done this to observe whether the light sensor can fit into the other port where she has put the sound sensor before (Line 18). However, Roy is upset with Hazel undoing his work, resulting in a physical altercation between the two which required intervention by a teacher (Line 17).

In this vignette, Das’s nudges for taking turns (Lines 8, 11) were mostly unsuccessful in reconciling conflicts between the pair. As Roy was heavily enthusiastic about robots, taking turns for KIBO was challenging for him; he frequently assumed a dominant play role. Conversely, Hazel desired to accomplish tasks independently and was unwilling to accept Roy’s attempts to take over. This illustrates how children’s contrasting personalities and interpersonal relationships may shape technology-mediated play and require mediation by trusted adults (e.g., teachers) to manage conflicts.

#### 6.3.3 Negotiating Turn Taking and Cooperation.

Despite intermittent conflicts around KIBO, over time children learned to negotiate turn taking, shared resources, and eventually went on to help one another. In the vignette in Table 11, both Bryce (GDD) and Shiloh (neurotypical) want to take the sound sensor but Bryce does not want to share (Line 1). However, with Das’s nudging, Bryce holds it up for Shiloh to see (Line 2). Shiloh then proposes a compromise: *“He can hold it. I can hold the next one”* (Line 4). Again, when Bryce expresses his interest to press the start button to make KIBO move, Shiloh negotiates for a chance to clap to trigger the sound sensor following Bryce’s button press (Lines 6–8). Finally, when Bryce tries to grab a lightbulb that Shiloh is holding, Shiloh asks him to hold the next one, knowing that there will be multiple lightbulbs available for both of them (Lines 11–14). In all three cases, the exact terms of resource sharing and turn taking are not always proposed by the adult, rather a child coordinates turn taking with minimal involvement (only approval) from the adult.

Table 12 presents an even stronger form of collaboration between Bryce and Shiloh where they are actively working together towards a shared goal of creating a new code. Both children exchange the connected sequence of blocks back and forth so that each can add new blocks to it (Lines 3–8). As the sequence gets longer, Bryce holds it in place while Shiloh attaches the End block to it (Line 9). Finally, they *“high-five”* to celebrate their joint effort (Lines 11–12).

Both Bryce and Shiloh carried forward this cooperative attitude while playing with other children. In a session with Eva (neurotypical), who was interacting with KIBO for the first time, Bryce said, *“I’ll help Eva! Eva doesn’t know how to do this.”* When Eva attempted to connect the motor and the wheel, Bryce drew her attention to the visual instruction guide and encouraged her, saying *“Yeah, put it together like that!”*

Likewise, Table 13 illustrates Shiloh assisting Wyatt (autistic, nonspeaking) in assembling KIBO. Encouraged by Das’s nudging, Shiloh demonstrates Wyatt how to attach a wheel to a motor and hands those to Wyatt (Lines 2–4). Wyatt, however, orients the pieces incorrectly and observes how Shiloh is connecting another set of motor and wheel (Line 5). With further probing from Das, Shiloh provides hand-over-hand guidance to Wyatt by connecting a wheel with a motor Wyatt is holding (Line 9). Next, Shiloh encourages Wyatt to try it himself (Line 10) and cheers when Wyatt starts rotating the wheel (Line 12). Later, Shiloh gives a sound sensor to Wyatt even without direct nudging from an adult, saying *“I’ll let Wyatt do it.”* Our encouragement for collaboration, coupled with the fact that Shiloh did not need to contest with Wyatt for resources (unlike with Bryce), might have motivated him to assume a supportive role. This cooperative play brought them a sense of joint accomplishment, as Shiloh exclaimed after decorating KIBO with pictures of their favorite objects and animals: *“Whoa, look how we made a robot with Wyatt’s squish-mallow and my giraffe!”*

Overall, neurodiverse children engaged in intermittent yet fluid interactions with each other, which ranged from competition and conflicts to reciprocal turn-taking and cooperation, sometimes without direct nudging by the adults. Since computational kits are commonly used in group settings rather than for solitary play (likely due to their cost) and are difficult to repair, these insights about how children amicably negotiated conflicts and assisted each other can inform future approaches to CT-centric inclusive social play.

## 7 Discussion

Drawing on our deployment of KIBO in preschool classrooms, we revisit ways to nurture computational thinking among neurodivergent children and what it means to create an inclusive culture of technology-mediated social play.

### 7.1 Fostering Computational Thinking (CT) among Neurodivergent Preschoolers

Our work joins that of others showing that programmable robots like KIBO can help instill CT among children as young as 3–5 years old [3, 54], including those who are neurodivergent. We corroborate the insights from Albo-Canals et al. [1] who found that 9–17 years old autistic children were able to assemble KIBO, attach blocks, and press the start button to make the robot move. Moreover, with our pre-planned and in-the-moment adaptations, several neurodivergent children exhibited understanding of the “powerful ideas” of CT [6]. We argue that such strategic and dynamic adaptations are essential to make computational kits accessible and enjoyable to neurodivergent children. Table 14 summarizes our adaptations, KIBO design features, and environmental factors that influenced children’s understanding of CT concepts.

Our adaptations align with Alper et al. [2]’s design principles for accessible computational technologies for children. Visual aids such as pictograms and step-by-step instructions worked as *ramps*, making it easier to perceive and convey information for minimally- or nonspeaking children who relied on visuals and gesture-based communication. Scaffolding by the teachers or Das worked as *ladders* that helped neurodivergent children attempt complex tasks like debugging and scanning. Incorporating children’s favorite materials enabled *frames of interest* that made the activities more appealing to children like Sierra, who decorated KIBO as her imaginative superhero. Teachers’ hand-over-hand guidance provided necessary *reinforced corners* for children with significant developmental delay to explore and manipulate KIBO components. Through this, some children expressed a sense of accomplishment, self-efficacy, and maker mindset [13], as evident by Hazel exclaiming *“Done!”* after she successfully placed a sound sensor on KIBO.

While the examples above are promising, our adaptations could not fully support some neurodivergent children’s unique needs. For instance, Walker, Sam, and Erin had minimal engagement with KIBO, possibly due to their limited attention spans (2–3 minutes) or other activities grabbing their interest more (e.g., Walker spent most of his “free choice” times on a sensory spinning chair).^3^ Wyatt, Aaron, and Kevin attended three or more sessions each and showed interest in tinkering with KIBO components but did not demonstrate understanding of higher-level computational concepts [1, 5, 25]. We anticipate that the learning curve of KIBO, although appropriate for neurotypical and some neurodivergent preschoolers, may have been too steep for these children. In the next section, we present directions for rethinking the design of computational kits and activities for these children who need support the most.

### 7.2 Reimagining the Design of Computational Kits and Practices for Inclusive Social Play

Our work rejects the deficit-oriented understanding that asserts that neurodivergent children prefer playing alone and are unable or unwilling to engage in cooperative exchanges with peers [32, 65]. Neurodivergent children are often more reactive to environmental sensory stimuli than social cues (e.g., eye gaze, facial expressions, or nonverbal gestures) [74]. Hence, their attempts to initiate or respond to playful overtures may be ignored or misinterpreted due to their atypical nature (e.g., repeated movements, echoing spoken words, staring at shiny objects, or fascination with certain materials) [74]. This misalignment may lead to neurodivergent children struggling with joint attention, emotional synchrony, and social reciprocity with neurotypical peers [14, 16]. Nevertheless, neurodivergent children desire social connections, shared experiences, and meaningful friendships through play [43, 50]. Our inquiry into the preschool classrooms is an attempt to investigate how social play among neurodiverse children unfolds through interaction with and around a computational kit. Here, we revisit our findings taking the lens of Wolfberg [73]’s Integrated Play Groups (IPG) model and enumerate design possibilities and practical guidance to enable mutually engaging activities for neurodiverse children, especially those whose needs are not fully met by existing computational kits.

#### 7.2.1 Recognizing Neurodivergent Expressions of Play.

IPG emphasizes nurturing neurodivergent children’s unconventional or ambiguous forms of play by recognizing every action and interaction—whether directed to oneself, peers, adults, or objects—as purposeful expressions of play [74]. Likewise, computational kits can be redesigned considering neurodivergent children’s unique proclivities. For instance, kits may incorporate everyday materials beyond wooden blocks or tiles [75], such as paper, textiles [48], or kinetic sand to help children like Aaron initiate play attempts, who had an affinity for fiddling with dirt. Sensors may be attuned to capture the sounds of banging or the motion of shaking objects as Avi’s intent to play. Whole-body gesture mechanism may be implemented to allow children to program the robot by enacting desired movements, like swinging or spinning as Walker enjoyed doing.

#### 7.2.2 Providing Audio-Visual Cues for Mapping Input to Output.

IPG calls for guiding play catered to children’s comfort level and competence and helping them move gradually beyond their present capacity [74] within their zone of proximal development [68]. In our study, some neurodivergent preschoolers struggled to grasp the alignment between KIBO’s input and output. For instance, they were confused about the purpose of the Begin and End blocks, since KIBO did not perform any observable actions corresponding to those. To remedy this, a future version of KIBO may start and conclude code execution with spoken announcements like ‘Begin’ and ‘End’. Further, to help children locate errors in the code, Das pointed to individual blocks in a code sequence while KIBO performed associated actions. In the future, coding blocks may provide real-time visual cues, such as lighting an LED embedded in the block whose action KIBO is performing. This may help children detect blocks that might have been missed during scanning (*debugging*), if the robot is waiting for an input e.g., clap to execute the rest of the code (*if-then conditionals*), or what blocks are being repeated for how many times (*loops*). Over time, children may get proficient in creating and debugging codes without direct audio-visual cues or adult assistance. Beyond this, computational kits should also include advanced hardware, for instance, sounds sensors responsive to soft claps by young children and sound output adjustable to the surrounding noise level.

#### 7.2.3 Scaffolding Play with Micro-tasks.

IPG highlights the fluid role of adults in scaffolding play [74] so that children can play according to their interests and strengths—irrespective of how sporadic or prolonged their engagement might be (Section 4.2.5). During our sessions, the teachers and the lead researcher, Das moderated activities by modeling actions and dialogue, assisting neurodivergent children when required, and encouraging neurotypical children to be preseverant when their neurodivergent playmates needed more time to accomplish tasks. Moving forward, we see opportunities for teachers to plan activities such that children with misaligned attention spans can work together more seamlessly. As the notion of “crip time” [33] suggests embracing a “flexible approach to normative time frames” [53] to align with individual differences, teachers may incorporate micro-tasks, allowing children to engage with computational kits for short bursts of time (2–3 minutes) and build off each other’s micro-tasks. Thus, children like Bryce may create a new code sequence, while someone like Wyatt may only assemble components and others like Aaron may press the start button to make a pre-programmed robot move. By performing these micro-tasks, neurodivergent children may develop a sense of accomplishment even if they do not have the bandwidth to finish a lengthy programming task all at once.

#### 7.2.4 Enabling Technology-Mediated Collaboration.

IPG recommends guiding social communication by preparing children to recognize, interpret, and respond to each other’s communication cues [74]. During our deployment, teachers oriented neurodiverse pairs to observe and mirror each other’s actions, maintain joint attention (e.g., watching together how KIBO executes a code), and perform joint action (reciprocal turn-taking or holding an object together); also see Section 4.2.4. Some of these collaborative actions could be mediated by technologies as well [9, 30, 52, 62]. For example, the robot could be preset to execute a code only when a pair of children simultaneously press the start button—an action that can be sensed by wearables worn by the pair. Coding blocks could be redesigned such that two blocks will connect only when the pair hold one block each, sensed by their wearables. Importantly, such technology-mediated collaboration needs to be contextualized and dynamically adjustable by adults rather than being solely enforced by the technology [62].

#### 7.2.5 Crafting Strategies for Conflict Resolution.

We noted recurrent conflicts arising between children around the ownership of KIBO, leading to negotiation for turn-taking and sharing resources—all of which are important facets of group work and social skills development. CT kits are relatively expensive, likely to break during physical altercations, and difficult to repair, which necessitates teaching children strategies to resolve disagreements constructively [23, 51]. To this end, we incorporated pretend play by encouraging children to decorate KIBO as their favorite characters and when conflicts arose, reminded them to handle KIBO with care to avoid “hurting” it. However, some neurodivergent children needed more assistance to manipulate KIBO, which engendered their neurotypical playmates’ misconceptions about their competence. While teachers clarified these misconceptions through verbal explanations, implementing character narratives [62] and expanded proxy [14, 41, 51] could also nudge neurodiverse groups to work through conflicts and develop mutual empathy [43]. More broadly, computational kits and activities should facilitate all stages of play [59], including onlooking, parallel play (i.e., playing side-by-side in the same space), and associative play (e.g., imitating peers and using similar materials). Through this, children with contrasting personalities like Roy and Hazel may get comfortable to play alongside each other before moving to a tightly-coupled play routine.

### 7.3 Limitations and Future Work

Future research could investigate a longer deployment of computational kits allowing neurodiverse children to explore CT concepts we could not cover within the time constraints allocated by our partner school, including patterns, loops, modularity, and designing complex projects (e.g., creating games or interactive stories). Additionally, future work could integrate strategies attuned to the needs of nonspeaking children (e.g., co-design beyond words [69]).

## 8 Conclusion

With a goal to promote inclusive approaches to teach CT, we situated our study within two preschool classrooms involving neurodiverse children. Building on interviews with teachers, we deployed a programmable robot KIBO for eight weeks in the classrooms. Our interaction analysis revealed that children enjoyed making and coding with KIBO, while engaging in cooperative and competitive play around the technology. We highlight how strategic and in-the-moment adaptations catered to children’s access needs and interests can facilitate CT. These insights encourage us to reimagine technology-mediated social play to cultivate equitable opportunities for children with diverse abilities and developmental phases.

## Figures and Tables

**Table 1:** Avi (autistic, nonspeaking) attaches the motor and the wheel with teacher T2’s help.

1		(Avi shakes a wheel and a motor as if weighing them.)
2	Das:	Do you want to put it here? (Points to the motor slot.)
3		(Avi places the wheel on the motor slot.)
4	Das:	That one. (Points to the motor Avi is holding) Put it here. (Points to the motor slot) That one. Put it in.
5		(Avi fiddles with the motor and the wheel; seems to try connecting those pieces but fails.)
6		(Avi bangs the motor with the wheel and brings them near his ear.)
	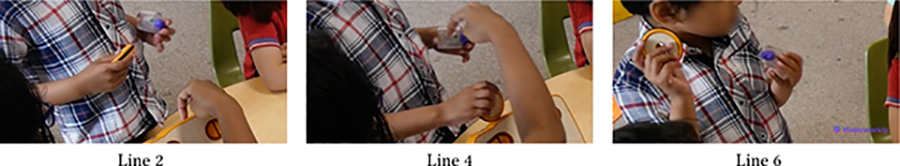	
		…
7	T2:	Can I help? (Holds out her hand towards Avi.)
8		(Avi gives T2 the motor and the wheel. T2 takes the motor and gives the wheel to Avi.)
9	T2:	(Intonates) I’m gonna push. (Inserts the motor into the slot but does not fully secure it.)
10		(Avi strikes the motor with the wheel in his hand. T2 takes the wheel away from him.)
11	T2:	Push. (Points to the motor inserted into the slot.)
12		(Avi takes out the motor from the slot and shakes it.)
	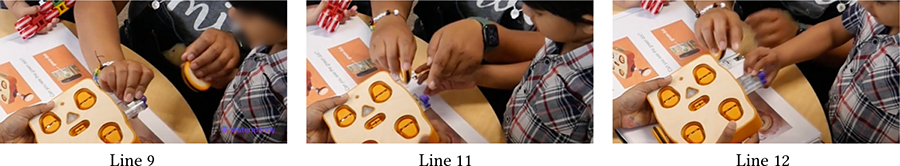	
13	T2:	That’s pull. (Takes the motor from Avi’s hand and inserts it again into the slot.) Let’s push… Push…
14		(Avi pushes the motor that T2 inserted into the slot.)
15	Das:	Good job!
16	T2:	(Grabs the wheel) Let’s put this in. (Places the wheel on the motor’s axle) Push.
17		(Avi pushes the wheel on the motor’s axle while T2 holds it in place.)
18		(T2 pushes the wheel tightly to secure it in place while Avi continues to hold it.)
	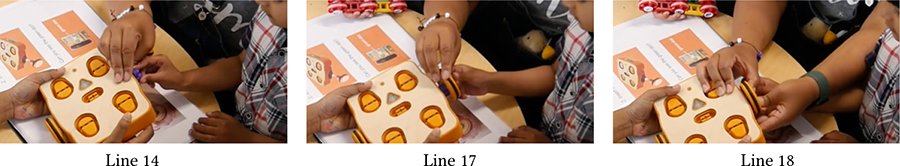	

**Table 2:** Sierra (autistic, minimally-speaking) decorates KIBO with her personalized character “Super Sierra.”

1	T1:	(Points to the superhero photo) Sierra, look! She has a cape, just like you!
2	Sierra:	Super Sierra!!!
3	T1:	Which one do you wanna tape? Super Sierra or bunny rabbit?
4	Sierra:	Super Sierra. (Pats on the superhero photo.)
		…
5	Sierra:	I wanna do it! (Places the whiteboard taped with the superhero photo on KIBO.) Yay!
6	Das:	There you go. Yayy! (T1, Das, and Sierra clap.)
		…
7	T1:	What should we name the robot? The robot’s name is…
8	Sierra:	Superhero.
9	Das:	Where does the Superhero live? (Sierra is distracted by pictures on a wall.)
10	T1:	Sierra, Superhero lives in the… (Pause) lives in the…
11	Sierra:	Sky!
12	Das:	What does the Superhero like to do? (Sierra does not respond.)
13	T1:	The Superhero likes to… (Pause) Jump on the trampoline?
14		(Sierra Shakes her head.)
15	T1:	Fly?
16	Sierra:	Yes! (excited)
	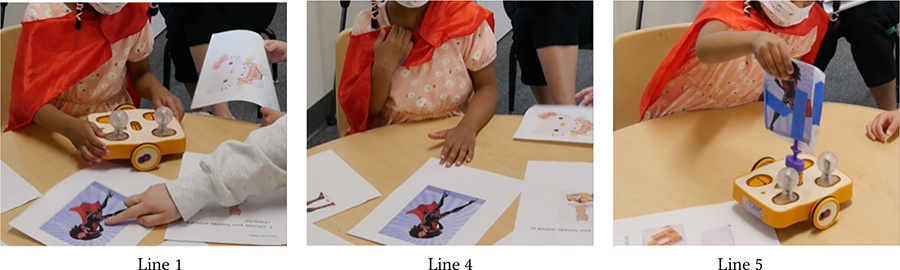	

**Table 3:** Violet (language delay) and Mila (neurotypical) select blocks to create a new code and explores the sound sensor.

1	Das:	Do you remember which block you used to begin your code? (Mila grabs the Begin block.
2	Das:	Violet, which block do you use to end your code? (Both move to grab the End block. Violet grabs it.)
3	Das:	Good job! How do you want to create a new program? Which block do you want to use?
4	Violet:	I want Beep. (Points to the Beep block.)
	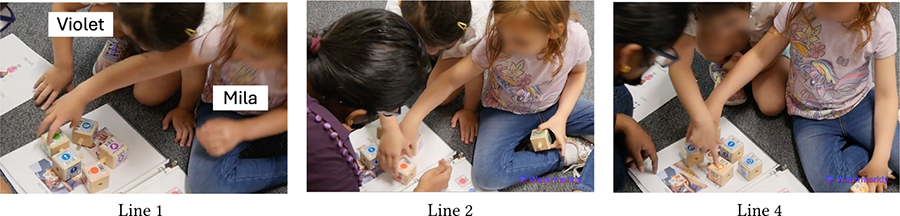	
		…
5	Das:	(Holds up the sound sensor) Like our ear, KIBO has something that it uses to hear sounds. What does it look like?
6	Mila:	An ear. (Grabs the sound sensor and places it on the ear image. Violet seems distracted.)
7	Das:	Violet, do you think it looks like an ear? (Violet nods.) You can say yes, no, maybe. (Violet points to the ‘yes’ image.)
8	Das:	Yes! And it can help KIBO listen to sounds, okay? (Violet nods.)
		…
9	Das:	Do you remember what you used to make KIBO listen to the sound?
10	Violet:	(Holds the sound sensor.) Ear!
11	Das:	And which block do you use to make it respond to the sound? (Violet smacks the Wait-for-Clap block.) That one!
	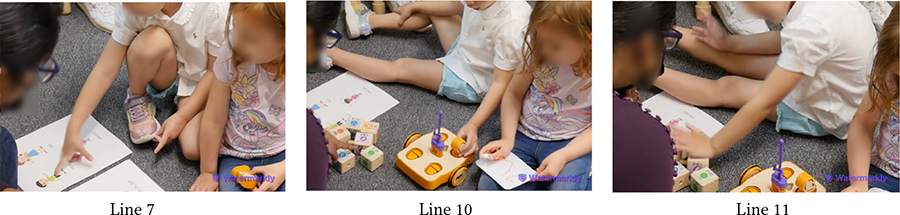	

**Table 4:** Sierra (autistic, minimally-speaking) successfully creates new code sequences on her own.

1		(Sierra reaches over to grab a block that was not connected to the code sequence.)
2	Das:	(Holds the new block) It’s for the white light. But we didn’t put it.
3		(Sierra grabs the block from R’s hand and takes out the End block from the sequence.)
4	Das:	You want to put it? Okay.
5		(Sierra turns around the Light block to orient it correctly and connects it with the sequence.)
6		(Sierra reconnects the End block.)
	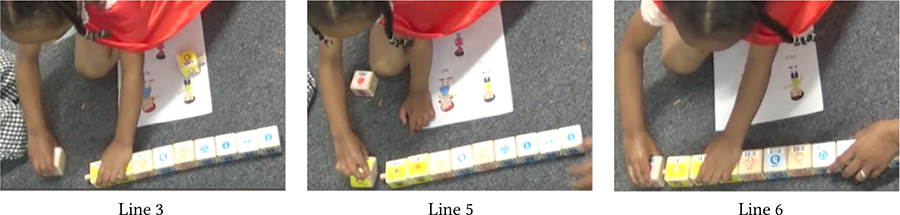	
		…
7	Das:	(Brings out four blocks.) These are our coding blocks, okay?
8		(Sierra connects the Begin block with Backward, then Forward, and finally the End block.)
9	Das:	What did you make here?
10	Sierra:	(Holding up the sequence) Begin, Backward, Forward, End.
		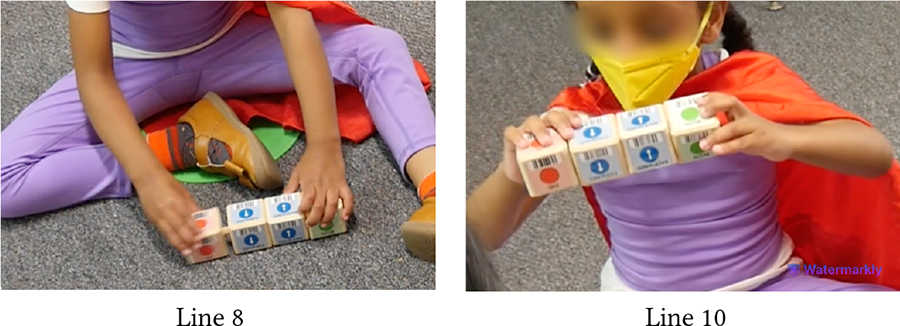

**Table 5:** Bryce (GDD) and Shiloh (neurotypical) identify that KIBO is waiting for clap.

1		(Shiloh presses the start button twice. Nothing happens.)
2	Das:	Why is it not doing anything when you are pressing? Bryce, Can you tell me?
3	Bryce:	Clap! (Starts clapping.)
4	Das:	Yes, it’s waiting for your clap. Clap closer to this. (Points to the sound sensor.)
5	Shiloh:	(Shiloh claps loudly near the sensor.) He’s waiting for your clap.
		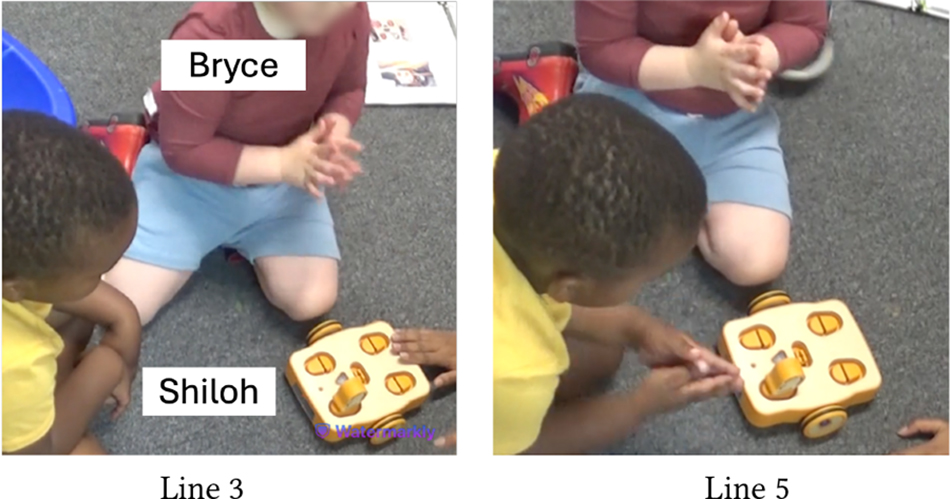

**Table 6:** Aaron (autistic, nonspeaking) claps and presses the start button (cause) to make KIBO move (effect).

1		(Aaron observes KIBO moving. Once it stops, he explores it.)
2	T7:	He wants to make it go again.
3	Das:	Oh, okay (Presses the start button and claps near the sound sensor. Aaron claps repeatedly.)
4	T7:	Good job clapping, Aaron! Ready? (Guides Aaron’s hands to clap near KIBO.)
5		(R claps loudly near the sound sensor. KIBO starts to move. Aaron observes attentively.)
6		(Once KIBO stops, Aaron presses the start button.)
	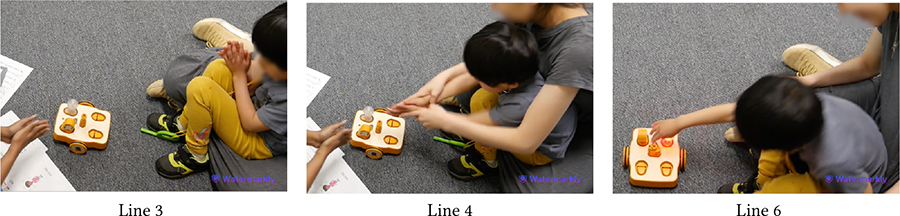	

**Table 7:** Cooper (autistic) and Hugo (neurotypical) determine which block action KIBO has skipped.

1		(Both children observe KIBO moving.)
2	Das:	Do you think KIBO did everything you asked it to do?
3		(Cooper hovers a finger over some blocks and then points to the Begin block.)
4		(Hugo points to the Begin block.)
5	Das:	It didn’t do this one? (Points to the Begin block). It begins your code, right?
6		(Cooper presses the start button again.)
7	Das:	(Points to the blocks one by one.) It goes backward, forward, shake, beep… red light on, white light on… What did it not do?
		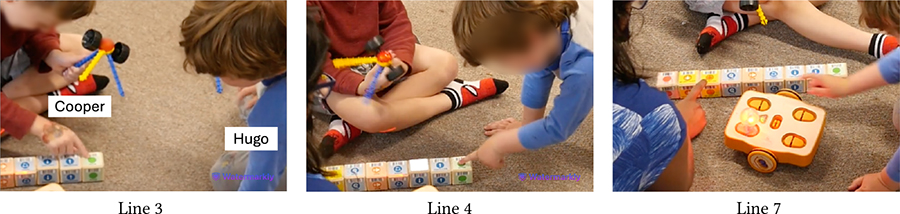
8	Cooper:	That one! (Points to the Beep block with another toy.)
9	Hugo:	That one! (Points to the Beep block.)
10	Das:	It didn’t do Beep? Let’s try again. (Hugo presses the start button.)
11	Das:	(Points to the blocks one by one.) Goes backward, forward, shake… (brings ear closer to KIBO) Beeeep… And then red light on, white light on.
12	Das:	Which one did it not do? (Pause) Did it do this one, did it spin?
13	Cooper:	No! (Shakes his head.)
		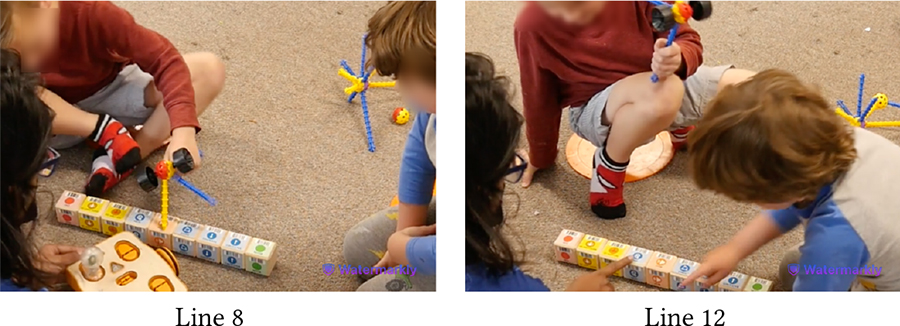

**Table 8:** Bryce (GDD) and Shiloh (neurotypical) discuss the hardware of KIBO.

1	Das:	(Holds up a wheel.) Do you remember what this is called?
2	Shiloh:	The arm. (Bryce inserts a motor into KIBO’s slot.)
3	Bryce:	KIBO! (Takes the wheel from Das and connects it with the motor he attached to KIBO.)
		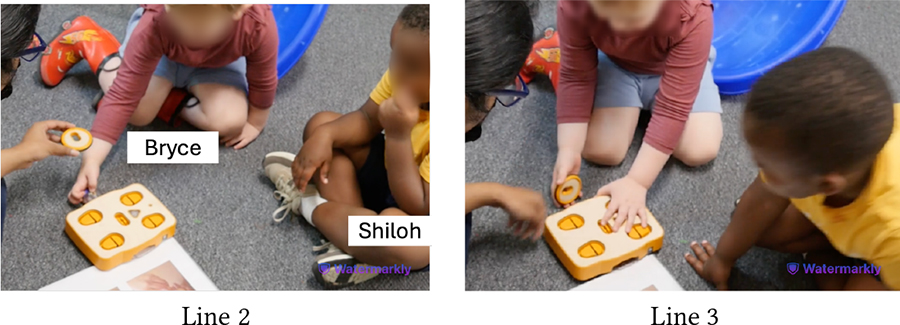
4	Das:	And what’s this piece? (Holds up another wheel.)
5	Shiloh:	A wheel.
6	Das:	And what is this? (Holds up a motor).
7	Bryce:	Motor.
8	Das:	What does the motor do?
9	Shiloh:	Spins. You have to put the circle in there (attaches the wheel to the motor.) I can even make a sound (Rotates the wheel, emitting a twisting sound. Then he inserts the motor into KIBO’s slot.)
10	Shiloh:	KIBO is the body (Points to KIBO) and these are the legs (Points to a lightbulb image.)
11	Bryce:	These are the legs. (Points to the wheel on KIBO.)
12	Shiloh:	Bryce Bryce, that is the wheel. (Points to the wheel)
		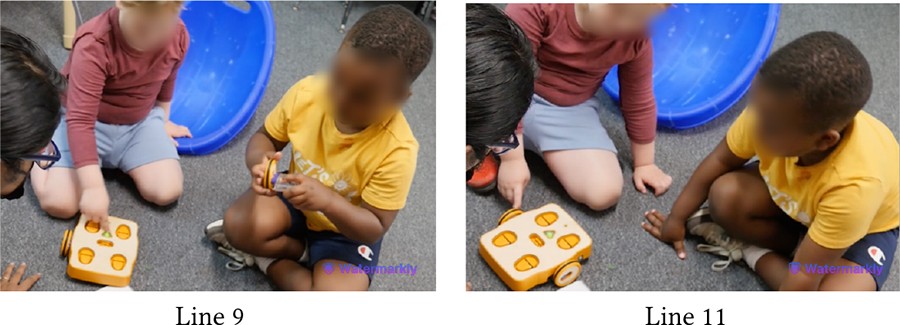
		…
13	Bryce:	KIBO is a car.
14	Shiloh:	I mean it’s a car robot.
15	Das:	Do you think KIBO can think on its own?
16	Shiloh:	No.
17	Das:	Why not?
18	Bryce:	It doesn’t work yet.
19	Das:	So how do you tell KIBO to do something?
20	Shiloh:	By scanning the blocks. And then when you scan the blocks, it will do the dance. (Mimics a dance move.)

**Table 9:** Teacher T2 encourages Owen (neurotypical) and Avi (autistic, nonspeaking) to work together.

1		(Avi shakes a motor and a wheel.)
2	Owen:	I need to grab that to put it together. (Points to the motor Avi is holding.)
3	T2:	(To Owen) You know he’s looking at it. The way he explores it–(shakes hand imitating Avi)–he’s trying to figure out what it’s made of.
4		(Avi tries connecting the wheel with the motor but after some time resumes shaking them.)
5	Owen:	And now give me! (Stretches out hand) Let me try it. He doesn’t know how.
		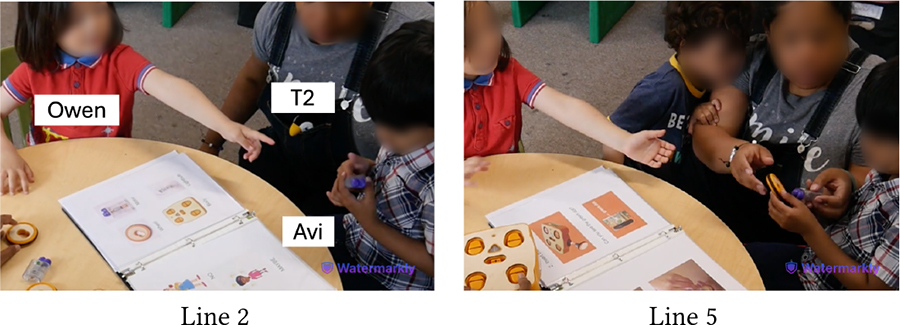
6	T2:	He needs help. I’m going to help him, okay?
7		(Avi leaves the wheel on the table. Owen reaches over to grab the wheel but T2 grabs it.)
8	T2:	You want to hold it with him? (Gives Owen the wheel.) Let’s do it together. (Holds Avi’s hand with the motor.)
9		(Owen takes the wheel and connects it to the motor Avi is holding.)
	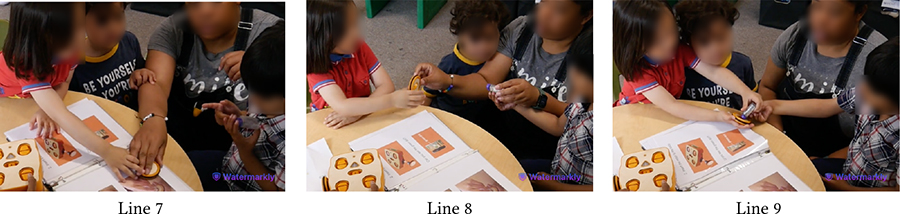	

**Table 10:** Hazel (Down Syndrome, minimally-speaking) and Roy (neurotypical) compete for KIBO components.

1		(Hazel tries to connect a lightbulb with the flagpole.)
2	Roy:	(Inserts another lightbulb on one of KIBO’s four ports.) I did it!
3	Das:	Hazel, let’s try putting it over here (points to an open port), like the way Roy did?
4		(Hazel places the lightbulb on the port Das gestured.)
5	Roy:	Actually it should go right here. (Roy takes out the lightbulb Hazel has placed and inserts it in another port.)
	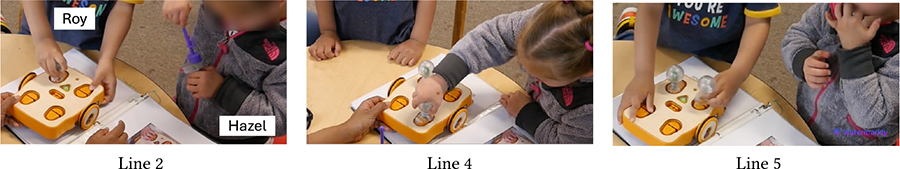	
6		(Hazel takes out the lightbulb. Roy doesn’t want to let it go. Hazel forces.)
7	Hazel:	(Pats her chest.) Me!
8	Das:	(To Roy) Okay. Okay. You had your turn. Let Hazel put it.
9		(Hazel puts back the lightbulb on the port where she has placed it before.)
10	Das:	Now what does this look like? (Holds up the sound sensor. Both stretch their hands to take it.)
11	Das:	You both can have your turn.
12	Hazel:	(Grabs the sound sensor and holds it away from Roy.) No! (Pats her chest.)
	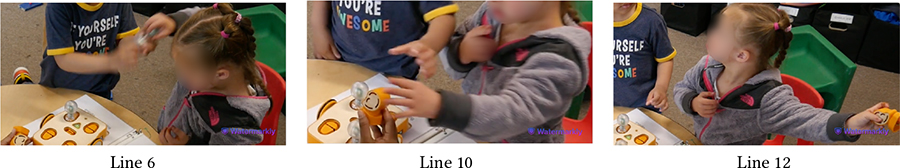	
13	Roy:	(Grabs the light sensor from R’s hand and inserts it in an open port.) I did that!
14	Hazel:	(Inserts the sound sensor in another port.) Done!
15	Das:	You made it!
16		(Hazel takes out the light sensor Roy has inserted. Roy reaches out to grab it. Hazel keeps it away from Roy.)
17	Roy:	Hey! (Roy grabs Hazel. Hazel shoves Roy. A teacher intervenes and releases their grip.)
18		(Hazel takes out the sound sensor she has put before and inserts the light sensor on that port.)
	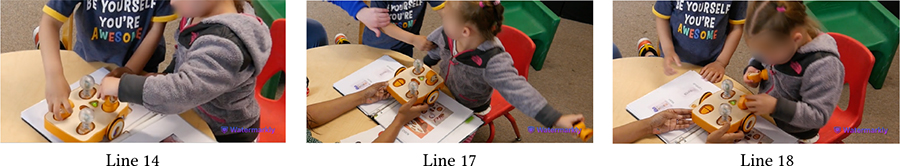	

**Table 11:** Shiloh (neurotypical) and Bryce (GDD) negotiate turn taking.

1		(Shiloh reaches over to take the sound sensor but Bryce holds it tightly. Shiloh retracts.)
2	Das:	(To Bryce) Show it to Shiloh. He wants to see it too. (Bryce holds up the sensor. Shiloh observes closely.)
3	Das:	(To Shiloh) You want to hold it?
4	Shiloh:	Umm, no. He can hold it. I can hold the next one.
5	Das:	OK, good job working together!
		…
6	Bryce:	Can I press the button?
7	Das:	Yes, you can… You will both get your turn.
8	Shiloh:	And after Bryce presses the button, can I clap?
9	Das:	Yes, absolutely!
		…
10		(The pair have attached blocks for white, blue, and red light to the sequence.)
11	Shiloh:	I think it’s gonna do three colors.
12	Das:	You have all these blocks, but what do you need to make the light?
13	Shiloh:	The light! (Grabs a lightbulb from R. Bryce tries to take it too but Shiloh keeps it away.)
14	Shiloh:	You can do the next one. (Bryce retracts.)
	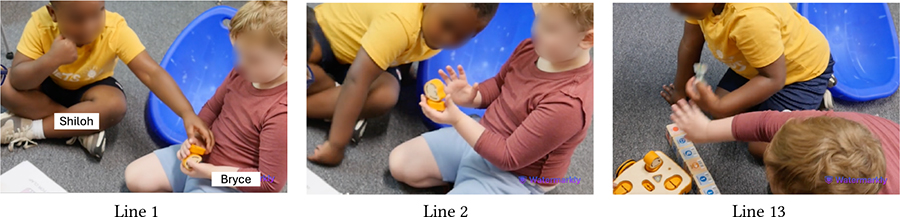	

**Table 12:** Bryce (GDD) and Shiloh (neurotypical) work together and celebrate joint effort.

1	Das:	Which block do you want to use next?
2	Shiloh:	I want to use this block. (Points to the Wait-for-Clap block.)
3	Das:	(Puts the Wait-for-Clap block in front of Bryce) Bryce, you can put it with the Begin block. (Points to the Begin block Shiloh is holding).
4	Das:	(Bryce hands Shiloh the Wait-for-Clap block.) Yes, work together.
5		(Shiloh connects the Wait-for-Clap block with the Begin block and hands those to Bryce.)
		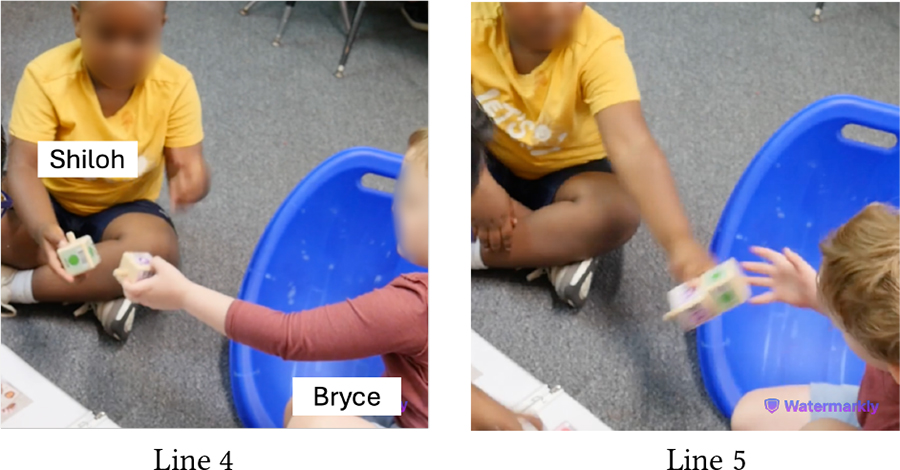
6	Das:	Bryce, which block do you want to use next?
7	Bryce:	This one. (Takes the Backward block and adds it to the sequence.)
8	Shiloh:	I want to use this one. (Grabs the Forward block and gives it to Bryce.)
9		(Shiloh connects the End block to the sequence while Bryce holds it.)
10	Das:	Good job working together.
11	Shiloh:	High five Bryce! (Stretches out hand.)
12	Bryce:	Yeah! (High-fives Shiloh.)
		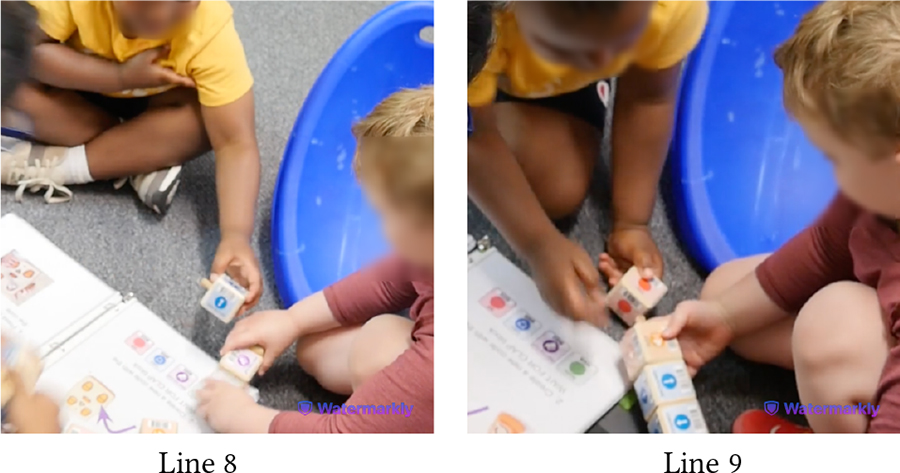

**Table 13:** Shiloh (neurotypical) helps Wyatt (autistic, nonspeaking) and cheers for him.

1		(Shiloh and Wyatt each take a wheel and a motor and tinker with them.)
2	Das:	Shiloh, do you want to show how to do this to Wyatt?
3	Shiloh:	Wyatt– (Holds up the motor and the wheel and shows how to connect those. Wyatt doesn’t look.)
4	Shiloh:	Now you try it, Wyatt. (Gives the motor and the wheel to Wyatt.)
5		(Wyatt tries to attach the non-axle side of the motor to the wheel. Then he observes Shiloh connecting another set of motor and wheel.)
		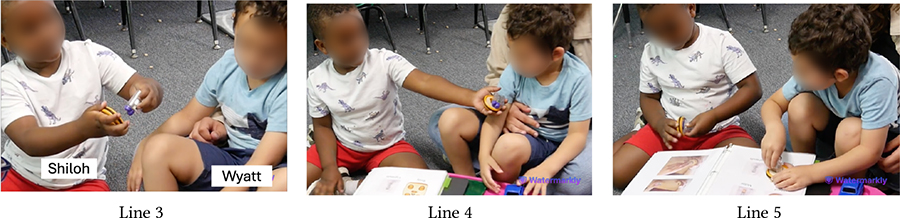
6	Das:	(To Shiloh) Perhaps you hold the wheel and Wyatt holds the motor. And then put it together.
7		(Shiloh takes the wheel from Wyatt. Wyatt continues fiddling with the motor. Shiloh holds the wheel in front of Wyatt.)
8	Das:	Wyatt, do you want to put it together like this? (Holds up a connected motor and wheel piece.)
9		(Shiloh connects the wheel to the motor Wyatt is holding.)
10	Shiloh:	Wyatt! (Holds up the connected motor and wheel) Do you wanna try? (Places the connected piece in front of Wyatt.)
11	T9:	Thanks Shiloh! (Wyatt takes the piece and rotates the motor attached to the wheel.)
12	Shiloh:	Go Wyatt! Go Wyatt!
		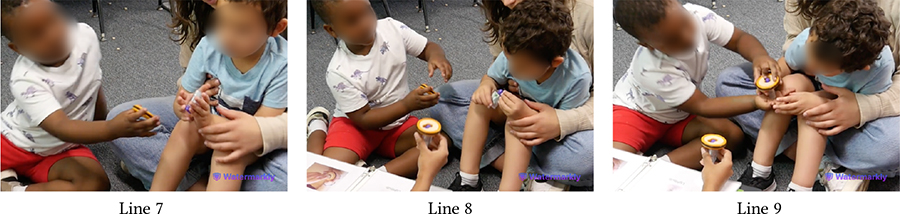

**Table 14:** Powerful ideas of CT [6, 54] learned by neurodivergent children and factors that impacted their learning.

Powerful idea	Examples from neurodivergent children	Factors that impacted learning
Representation	Bryce, Violet, Sierra, and Cooper understood that different coding blocks translate to a unique KIBO action.	Hindered: Lack of one-to-one mapping between Begin and End blocks and their actions. Helped: Visual aids we created (printed pictograms), KIBO’s default features (colors, shapes, and symbols of coding blocks, sound and light sensors resembling ears and eyes).
Algorithms (sequencing)	Bryce, Violet, Sierra, and Cooper created code sequences to make KIBO dance Hokey Pokey and perform other actions.	Helped: Visual aids we created (printed step-by-step instructions with images).
Control structures (cause and effect)	Bryce, Violet, Sierra, and Cooper understood if-then conditional, i.e., clapping will trigger the sound sensor. Aaron and Hazel understood that pressing the start button will make KIBO move.	Hindered: Limited responsiveness of the sound sensor. Helped: Adult scaffolding (making a loud clap for the sensor to get triggered).
Debugging	Bryce, Sierra and Cooper understood that a code sequence needs to be tested to check if KIBO performs all the coded actions, although they could not fully identify errors.	Hindered: Loud noise in the classroom. Helped: Adult scaffolding (pointing at the corresponding block when KIBO runs a code, vocalizing the Beep sound to overcome noise, giving concise options for probable errors).
Hardware/ software	Bryce, Sierra, and Cooper understood KIBO cannot work without scanning a code. Sierra and Bryce attempted scanning on their own. Hazel and Avi could attach motors and wheels and insert lightbulbs and sensors into KIBO’s ports.	Helped: Adult support for complex tasks like scanning, teachers’ hand-over-hand guidance (for Hazel and Avi).

## CCS Concepts

Human-centered computing → Empirical studies in accessibility; Collaborative interaction.

## Footnotes

1 We use the term *neurodiverse* to describe heterogeneous groups that include children who have typical (i.e., *neurotypical*) and children who have atypical (i.e., *neurodivergent*) neurocognitive functioning.

2 As recommended by the teachers, we use ‘nonspeaking’ instead of ‘nonverbal’ to recognize that children are able to communicate words in various ways other than speech [77], e.g., using AAC devices, visual cues, and gestures.

3 Avi attended only one KIBO session mainly because he was absent from the class during our scheduled activities on most days.

## A Session Plan and Participants

**Table 15:** Session plan for a 4-week deployment of KIBO in the neurodiverse preschool classrooms, adapted from [7].

Lesson	Activities	Objectives: Children will be able to…
1. Meet and decorate KIBO	Is it a robot or not? Meet KIBO parts Decorate KIBO as you wish	Compare robots with non-robots Attach wheels, motors, and lightbulbs to KIBO body Decorate KIBO as their favorite character
2. Dance Hokey Pokey	Meet the coding blocks Be a coder Code the Hokey Pokey dance	1. Recognize the Begin, End, motion, and light blocks 2. Understand that order matters when making a code 2. Understand that KIBO runs when the start button is pressed
3. Make it wait for clap	Compare human senses to robot sensors Meet the sound sensor Write a code including the Wait-for-Clap block Check if KIBO has done everything you coded	Understand that the code will need the sound sensor to listen to the sound input (clap) and run Identify if and where problems exist in a code
4. Put it all together	Plan for a new code using blocks Write a code including the Wait-for-Clap block Check if KIBO has done everything you coded	Understand the meaning of the blocks Understand that the code will need the sound sensor and the sound input (clap) to run Identify if and where problems exist in a code

**Table 16:** Details of children who attended the deployment sessions in two preschool classrooms. Children marked with a * used core wordboard, AAC apps on tablets, or visual cues for communication. Since we prioritized having at least one neurodivergent child in every session and the classes had more neurotypical children, some neurotypical children got to attend fewer sessions than the median (i.e., 3 in Class A and 2 in Class B). # Sessions: Number of sessions attended. Time: Total time spent with KIBO.

Class	Pseudonym	Age (Gender)	Neurodivergent conditions	Teacher-reported and observational notes	# Sessions	Time (min)
	Wyatt*	4 (M)	Autism, nonspeaking	Loves playing with shapes	4	68
	Aaron*	5 (M)	Autism, nonspeaking	Loves playing with dirt; working on verbal communication	3	48
	Walker*	5 (M)	Autism, nonspeaking	Loves spinning and a famous city landmark; limited attention span for tasks; working on communication skills	1	12
	Sierra*	5 (F)	Autism, minimally-speaking	Loves superheroes; can read; needs clear schedules and options for tasks	4	106
	Bryce	4 (M)	Global developmental delay	Working on taking directions from less familiar adults and being flexible	7	149
A	Violet	4 (F)	Language delay	Generally quiet	3	71
	Seth*	4 (M)	–	Some speech difficulties; going through speech evaluation	4	107
	Liam	5 (M)	–	Loves robots; wanted to have KIBO at home	3	55
	Shiloh	5 (M)	–	–	5	108
	Jasper	5 (M)	–	–	3	86
	Caleb	4 (M)	–	–	4	84
	Mila	4 (F)	–	–	3	64
	Eva	3 (F)	–	–	3	60
	Joel	4 (M)	–	–	2	27

	Sam*	5 (M)	Autism, nonspeaking	Gestalt language processor; loves songs, moving objects, shapes, Sesame Street, and quieter environment	2	46
	Avi*	5 (M)	Autism, nonspeaking	Gestalt language processor; likes to read; explores objects by shaking	1	21
	Cooper	5 (M)	Autism	Working with occupational therapist outside classroom	3	54
	Hazel*	5 (F)	Down Syndrome, minimally-speaking	Likes solitary play; Good at taking directions from adults	3	93
	Kevin*	4 (M)	Global developmental delay	Loves singing; working on expressive language	3	55
B	Erin*	4 (F)	Multiple disabilities	Screams for expression and getting attention; likes solitary play	2	44
	Billy	5 (M)	–	Some speech difficulties; going through speech evaluation; bilingual	4	86
	Roy	3 (M)	–	Loves robots; learning to participate in social play; bilingual	5	142
	Zoe	4 (F)	–	Interested in new things and helping	6	135
	Dylan	5 (M)	–	Loves numbers and maths	2	42
	Hugo	4 (M)	–	Likes to build; active imagination	2	37
	Owen	4 (M)	–	–	2	51
	Chloe	3 (F)	–	–	3	58
	Hana	4 (F)	–	–	2	50
	Kieran	5 (M)	–	–	1	16
